# Developing Consensus in the Assessment and Treatment Pathways for Autoimmune Encephalitis in Child and Adolescent Psychiatry

**DOI:** 10.3389/fpsyt.2021.638901

**Published:** 2021-03-29

**Authors:** GenaLynne C. Mooneyham, Vladimir Ferrafiat, Erin Stolte, D. Catherine Fuchs, David Cohen

**Affiliations:** ^1^National Institutes of Health, National Institute of Mental Health, Bethesda, MD, United States; ^2^Child and Adolescent Psychiatric Unit, URHEA, CHSR Sotteville les Rouen, Rouen, France; ^3^Department of Child and Adolescent Psychiatry, CHU Charles Nicolle, Rouen, France; ^4^Department of Psychiatry, University of Alberta, Edmonton, AB, Canada; ^5^Division of Child and Adolescent Psychiatry, Department of Psychiatry and Behavioral Sciences, Vanderbilt University Medical Center, Nashville, TN, United States; ^6^Department of Pediatrics, Vanderbilt University Medical Center, Nashville, TN, United States; ^7^Department of Child and Adolescent Psychiatry, Hôpital Pitié-Salpêtrière, AP-HP, Sorbonne Université, Paris, France; ^8^CNRS UMR 7222, Hôpital Pitié-Salpêtrière, AP-HP, Institut des Systèmes Intelligents et Robotiques, Université Pierre et Marie Curie, Paris, France

**Keywords:** autoimmune encephalitis, neuroimmunology, catatonia, psychosis, electroconvulsive therapy, neuropsychiatric symptoms

## Abstract

Children with a diagnosis of Autoimmune Encephalitis (AE) frequently require multi-disciplinary care in order to mobilize the assessment and treatment necessary for recovery. Institutional and provider practice differences often influence the diagnostic workup and treatment pathways made available to patients. There are a variety of provider coalitions in pediatric rheumatology, internal medicine, and neurology that have been making meaningful progress toward the development of consensus in assessment and treatment approaches to patient care. However, child psychiatry is currently underrepresented in this work in spite of the high psychiatric symptom burden seen in some young patients. The need for consensus is often made visible only with inter-institutional dialogue regarding patient care trajectories. We aim to review key updates in the assessment and treatment of children and adolescents with autoimmune encephalitis during the acute phase, with or without catatonia, and to outline provider perspectives by comparing current treatment models in the United States, Canada, and Europe.

## Introduction

Children and adolescents who present with new onset psychosis or catatonia may quickly be triaged to psychiatry for care regardless of whether their symptoms are etiologically associated with a physical or mental health condition. The differential for first episode psychosis is broad and may include genetic syndromes, inborn errors of metabolism, autoimmune diseases, tumors, and nutritional disorders to name a few (1). Our knowledge base regarding organic causes of both psychosis and catatonia has expanded greatly since the discovery of N-Methyl-D-Aspartate receptor (NMDAR) antibodies which cause autoimmune encephalitis (2–4). Autoimmune Encephalitis (AE) is an antibody mediated self-propagating inflammatory condition of the central nervous system and may present with a wide array of psychiatric symptoms to include but not limited to psychosis, mania, hypomania, catatonia, autistic regression, cognitive decline, and other conditions (4).

It is important to note that AE may be associated with a variety of antibodies and new antibodies are still being discovered in a dynamic fashion. One such example is the Myelin Oligodendrocyte Glycoprotein (MOG) antibody which is often associated with an acute disseminating encephalomyelitis (ADEM) but may also be associated with optic neuritis and encephalopathic features consistent with AE. The MOG antibody only became commercially available for testing within the last few years (5). At the time of this publication autoantibodies attacking the NMDA receptor, the MOG receptor, and the Glutamic Acid Decarboxylase (GAD) receptor are the most common antibodies found in pediatric patients with AE symptoms (6). There are epidemiological and etiological differences between the adult and pediatric patient populations with AE. Adult patients are more likely than children to have autoantibody production because of a paraneoplastic process. Another example is the VGKC (voltage gated potassium channel) antibody. Positive VGKC antibodies alone are not diagnostic for AE and binding to the LGI1 (leucine-rich-glioma-inactivated) and CASPR2 (contactin-associated protein-like) subunits allows for greater sensitivity and specificity (7). Children may have VGKC antibodies without AE (8) but the LGI1 and CASPR2 are rarely found in pediatric patients (7–10). This is a notable epidemiological difference of unclear significance.

The work-up offered to patients with an acute to subacute onset of neuropsychiatric symptoms can be highly variable. In October of 2019 we created an Autoimmune Encephalitis Special Interest Group (AE SIG) at the American Academy of Child and Adolescent Psychiatry 2019 Annual Meeting held in Chicago, Illinois, USA. This group remained in contact throughout the year from 2019 to 2020. In October of 2020 we mobilized a follow up forum through the American Academy of Child and Adolescent Psychiatry 2020 Annual Meeting which was converted to a virtual event due to the COVID 19 pandemic. The primary goal at both events was to ensure bidirectional information sharing and to create a space for open dialogue regarding international clinical practice norms. Providers were self-identified as being actively involved in the treatment of patients with AE and they were given the opportunity to describe resources available based on their geographic location. We worked to outline and acknowledge barriers to care and opportunities for growth in the assessment and treatment pathways made available to patients diagnosed with AE.

## Consensus in Nomenclature

A PubMed literature review was completed using the search terms “Autoimmune Encephalitis” AND “guidelines” which yielded 49 results. A second query was completed using the search terms “Autoimmune Encephalitis” AND “diagnostic criteria” yielding 34 results. Papers were reviewed and a filter of publication within the last 5 years was added limiting the results to 42 and 27 papers, respectively. Case series and case reports were later excluded. Many papers included in this search commented on the need for a standardized approach to patient care. Here we highlight two multi-disciplinary consensus publications.

Graus et al. published a sentinel article in 2016 outlining the diagnostic criteria for adults with AE (11). Diagnostic categories of “possible,” “probable,” and “definitive” AE are defined. The authors emphasize the importance of early diagnosis and early treatment as key variables in the trajectory of disease. Given the anticipated delays in diagnostic testing (often 7–10 days) and the potential influence on patient outcomes, readers are encouraged to consider first line interventions for patients with possible AE while awaiting the formal autoantibody results. The potential for patients to have negative antibody panels while still meeting clinical criteria for the disease model (i.e., seronegative AE) is acknowledged in this work. Providers have also treated pediatric patients by extrapolating from the Graus criteria.

In 2020, Cellucci et al. published a complimentary article outlining the clinical approach to the diagnosis of AE within the pediatric population (6). This article was written by a multi-disciplinary work group (neurology and rheumatology) initially formed in 2014 with the goal of outlining expert consensus opinion in patient care. Providers are encouraged to consider alternative diagnoses and to avoid prolonged use of immunomodulatory interventions when antibody testing and other paraclinical markers are negative. There is an intentional emphasis on clarifying when the category of “possible” autoimmune encephalitis is no longer an appropriate diagnostic term as data is collected/reported. A high impact visual algorithm is included as a quick reference for provider education.

## International Approaches to Assessment and Treatment Options

Autoimmune Encephalitis is a disease process with a constellation of clinical features that span several domains. This often includes changes in cognition, language impairments, movement disorders, seizures, and sleep disturbances. Multidisciplinary care requires consideration of both the antibody targeting the brain and management of the resulting clinical symptoms (11, 12). Rheumatology and Neurology work groups have recognized the need for standardizing assessments and immunomodulatory treatments (13). However, there is also a growing need for consensus amongst psychiatrists in treatment planning. For example, symptoms of catatonia may be present within any subtype of AE but is most commonly reported in NMDAR AE (11, 12). ECT may become a life-saving intervention when features of malignant catatonia occur. High dose benzodiazepines and/or ECT (electroconvulsive therapy) are well-established treatment modalities for catatonia (12) (with or without autonomic instability) but may be poorly understood by non-psychiatrists. The American Academy of Child and Adolescent Psychiatry set forth practice guidelines in 2004 including ECT as a safe and effective first line treatment for catatonia if symptoms are severe, persistent, and significantly interfere with function to include risk of death if not rapidly treated (14).

ECT treatment is also supported by a recent systematic literature review in both adults and youth, which found complete (60%) or partial (33%) neurologic recovery in 30 cases of anti-NMDA receptor encephalitis complicated by severe psychiatric symptoms, primarily catatonia (15). Likewise, the review by Tanguturi et al. also outlines the potential benefits of ECT in NMDAR encephalitis (16). They propose that a treatment algorithm for NMDAR encephalitis should include both benzodiazepines and/or ECT in order to manage catatonia as an augmentation strategy to the use of immunomodulatory medications. The authors noted the need for consistent guidelines for ECT and the use of established tools to measure response. They also acknowledged the variability of ECT availability in part due to stigma (16).

Insurance barriers to care can also influence access to diagnostic testing. As an example, ^18^F Fluorodeoxyglucose positron emission tomography (FDG-PET) has been emerging in the literature as having improved sensitivity, with abnormalities seen earlier on in the illness course (17–19). Access is dependent on the institution, and it has not yet been included in any proposed diagnostic criteria or assessment algorithms making efforts to obtain insurance prior authorizations particularly challenging. Nonetheless, this modality shows promise in cases which are seronegative yet remain suspicious for an immune etiology.

### Access to Care

Consultation and Liaison psychiatrists may be tasked with advocating for the multi-disciplinary work up needed to ensure an accurate diagnosis (20). A survey was created online using Microsoft Forms and sent to all members of the list serve for the AACAP SIG on AE and additional invited members of the AACAP Physically Ill Child Committee who are actively providing patient care for children and adolescents diagnosed with AE. Recipients were instructed to coordinate within their institution to ensure that only one representative of the identified program submitted a survey response. Time limit for completion was set at 48 h. Twelve questions were included and addressed location, access to imaging modalities, diagnostic procedures, and multidisciplinary consultants. We also queried access to intravenous immunomodulatory treatments, plasmapheresis, monoclonal antibody treatments, and ECT. A 42% response rate was achieved using convenience sampling as outlined above (*n* = 20) and the results were compiled using descriptive statistics (%) (see Table 1). In the US we looked at patterns by region (Southeast 4, Northeast 5, Midwest 2, West 1, and 1 unspecified). Single provider responses were also received from Europe, the Middle East, and from South America.

**Table 1:** International survey of AACAP Autoimmune Encephalitis Special Interest Group Members.

**Responses as % *N* = 20**	**US Southeast (4)**	**US Northeast (5)**	**US Midwest (2)**	**US West (1)**	**US Unspecified (1)**	**Canada (4)**	**Europe (1)**	**Middle East (1)**	**South America (1)**
MRI	100	100	100	100	100	100	100	100	100
Ultrasound	100	100	100	100	100	75	100	100	100
PET	75	80	100	100	100	50	100	0	0
VEEG	100	100	100	100	100	100	100	100	0
LP	100	100	100	100	100	100	100	100	100
Peds Rheum	100	60	100	100	100	100	100	100	100
Peds Neuro	100	100	100	100	100	100	100	100	100
Peds Crit Care	100	100	100	100	100	100	100	100	100
Peds ID	100	80	100	100	100	100	100	100	0
IVIG	100	100	100	100	100	100	100	100	0
IV Steroids	100	100	100	100	100	100	100	100	100
Plasmapheresis	100	80	100	100	100	100	100	100	0
RTX	100	100	100	100	100	100	100	100	0
TCZ	50	100	100	100	100	50	100	100	0
ECT Adolescent Yes/Maybe/No	75/0/25	80/20/0	50/0/50	0/100/0	0/0/100	50/50/0	100/0/0	0/0/100	100/0/0
ECT Child Yes/Maybe/No	25/25/50	40/20/40	50/0/50	0/0/100	0/0/100	50/25/25	0/0/100	0/0/100	100/0/0

*AACAP, American Academy of Child and Adolescent Psychiatry; ECT, electroconvulsive therapy; IV, intravenous; IVIG, intravenous immunoglobulins; LP, lumbar puncture; MRI, magnetic resonance imaging; Peds Crit Care, pediatric critical care; Peds ID, pediatric infectious disease; Peds Neuro, pediatric neurology; Peds Rheum, pediatric rheumatology; PET, positron emission tomography; RTX, rituximab; TCZ, tocilizumab; US, United States; VEEG, video electroencephalogram*.

Diagnostic resources were relatively consistent with the main variability being in Positron Emission Tomography (PET) available to only 14/20 (70%) of programs despite an increased interest in the use of this modality for diagnostic clarification as noted above (19). Only one program reported lack of access to video EEG (South America) and all providers indicated that it was possible to facilitate a lumbar puncture (LP).

Consultant availability was 100% for pediatric neurology, but only 60 and 80% of the northeastern US responders reported respective access to pediatric rheumatology (three states) and infectious disease (four states). South America reported absence of infectious disease consultants.

Treatment availability varied. All programs reported access to IV steroids. All US, Canadian, and European programs reported access to IVIG but one program (South America) reported no access to IVIG. All except South America have access to Rituximab while Tocilizumab is less consistently available (unavailable in two of the southeastern states, two Canadian programs, and not available in South America). Plasmapheresis was reported as available to all programs except one northeastern state and South America. Electroconvulsive therapy (ECT) is the most inconsistently available treatment both for child patients as well as adolescent patients. This is important to acknowledge given that malignant catatonia may be responsive to treatment yet can be fatal if unrecognized and/or untreated (14–16, 21).

**Figure 1:**
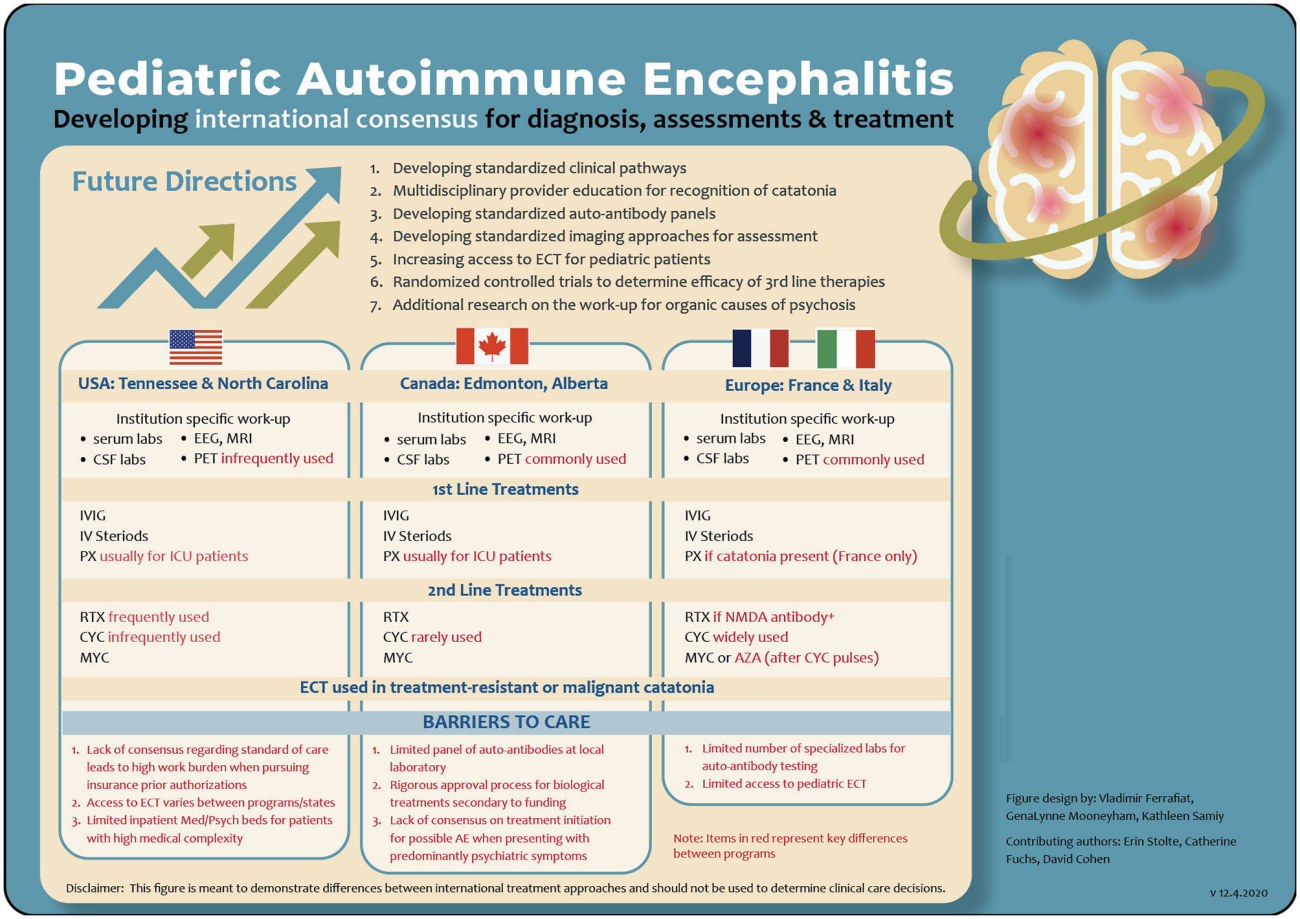
A comparison of program specific assessment and treatment pathways for AE. AE, autoimmune encephalitis; AZA, azathioprine; CSF, cerebral spinal fluid; CYC, cyclophosphamide; ECT, electroconvulsive therapy; EEG, electroencephalogram; ICU, intensive care unit; IV, intravenous methylpredinisone; IVIG, intravenous immunoglobulins; MRI, magnetic resonance imaging; MYC, mycophenolate mofetil; NMDA, N-methyl-D-asparate; PX, plasmapheresis; PET, positron emission tomography; RTX, rituximab.

Here we highlight a sampling of current treatment models across the globe (see Figure 1):

### A Canadian Treatment Model

The Canadian model varies across institutions with most AE cases managed by neurology or pediatrics and supported by consultant psychiatrists. We describe one model of tertiary care in Edmonton, Alberta. Patients with severe psychiatric symptoms, early negative investigations, and minimal neurological findings or seizures are managed on the psychiatric unit with consultation support by neurology and other subspecialties.

Atypical clinical features suspicious for AE, such as subacute onset of either working memory deficits, altered mental status, a new focal neurological finding or seizure would trigger an AE work up once alternative causes had been reasonably excluded. Initial work up includes serum studies, urine studies, a respiratory viral panel, serial EEG, and MRI brain. Lumbar Punctures are obtained by anesthesiology in the ECT suite. Standardized rating scales, such as the Montreal Cognitive Assessment and the Bush Francis for catatonia are utilized and repeated throughout the duration of a patient's hospitalization.

The Edmonton zone has relatively easy access to FDG-PET imaging. This group has observed several cases which were only FDG-PET positive yet responded well to immunotherapy. More research will need to be done to determine false positive rates and any potential overlap in features with psychiatric illness.

Immune treatments of IVIG and IV methylprednisolone are commonly used as first line treatments in stable patients and can be administrated on the psychiatric unit with appropriate staff training and support. Plasmapheresis is reserved for more severely deteriorating individuals requiring an ICU level of care. Most individuals receive second line treatment with Rituximab; however, it has become increasingly difficult to obtain approval for seronegative cases. Mycophenolate, cyclophosphamide, and bortezomib are rarely used. Tocilizumab does not yet have approval for AE but is available for other indications. ECT is used for cases with prominent catatonia not responsive to first line catatonia treatments. It has also been used for symptomatic treatment of severe mood and psychotic symptoms in AE with good success in a small number of individuals who failed to respond to medication management.

### US Treatment Models

One academic tertiary care center in the southeast (US) developed a Clinical Practice Guideline (CPG) (22). When AE is in the differential, consultations are obtained from child psychiatry, rheumatology, neurology, infectious disease, critical care, and the pediatric hospitalist team. The CPG outlines the assessment and treatment approaches with emphasis on monitoring change. This has enabled more effective communication between specialties. The CPG also defines a decision tree that emphasizes a consistent approach to the use of ECT in AE.

At another academic program in the southeast (US) psychiatry is directly embedded in an outpatient Pediatric Autoimmune Brain Disorders clinic and was, to our knowledge, the first program to provide this type of integrated psychiatric care for patients with AE (23). Patients are referred to this tertiary care academic institution from across the US and overseas. A multi-disciplinary team reviews all clinic referral packets and acceptance is based on availability and the degree of clinical suspicion for AE.

The multi-disciplinary team currently includes a Nurse Practitioner from pediatric rheumatology, a Nurse Coordinator, a Child and Adolescent Psychiatrist, a Pediatric Neurologist, and a Pediatric Rheumatologist. Families are seen during a single multi-disciplinary intake appointment with all care team members physically present in the room throughout the entirety of the clinical encounter. If clinically indicated, some patients are offered a more robust workup for autoimmune encephalitis and a portion of these patients require immediate inpatient care. The multi-disciplinary team members then continue seeing these patients in a consultation model throughout the hospital experience. Other families are offered outpatient testing to expand the diagnostic workup, when indicated, in connection with their local providers.

Standard and expanded labs from both serum and CSF are routinely completed and the possible workup is outlined in a 2017 publication (20). Ambulatory and/or video EEG as well as MRI are regularly offered by neurology, but PET is infrequently utilized at this time. Psychiatry provides medication management recommendations, bedside neurocognitive assessments, and clinical exams centered on the evaluation of catatonia when applicable. Rheumatology primarily manages the immunosuppressive medication regimens. First line therapies include IVIG and IV methylprednisolone. However, these are often used as once monthly bridge therapies while awaiting time to impact when other immunomodulatory medications are given. Rituximab is frequently utilized as a second line treatment particularly when a known autoantibody is identified. There is a growing number of patients for whom Tocilizumab has been found to be efficacious even after failure to respond to other therapies (24–26). Plasmapheresis is available as a first line therapy but is used infrequently and more often reserved for patients with severe autonomic instability who require an ICU level of care. Mycophenolate is an oral immunosuppressive agent that can be used as a solo intervention or an augmentation strategy, when clinically appropriate, given the ability to start and stop therapy in a dynamic fashion if there are concerns for recurrent infections or when a known surgical intervention is being planned. Cyclophosphamide is used judicially given the concerns for impact on fertility and the toxicity profile.

Collaborative relationships with Speech Therapy, Physical Therapy, Occupational Therapy, Neuropsychology, Radiology, and Neuro-Ophthalmology are also well-established and frequently utilized. A clinical guideline for mobilizing inpatient ECT was developed for use in patients with treatment refractory catatonia (27).

Given the current emphasis on collaborative and integrated care models, multi-disciplinary treatment teams are now gaining popularity within the US.

### European Treatment Models

One European network involves French academic teaching hospitals and an Italian institution specialized in the management of pediatric AE. This provider network has an evolving expertise in managing neuropsychiatric autoimmune conditions. Clinical centers in this network use a multidisciplinary and systematic assessment approach for patients with possible AE. Neurological examinations, a medical workup, neuropsychological assessments, and psychiatric evaluations are completed. Catatonia is monitored using the pediatric catatonia rating scale, a modified version of the Bush-Francis catatonia rating scale validated for the pediatric population (28).

The public health care system in Europe provides relatively quick access to diagnostic testing and potential treatments regardless of their cost. The identification of clinical red flags and a standardized laboratory workup (both serum and CSF) are used in combination with an immunosuppressive treatment challenge even in cases without paraclinical marker positivity in the initial data gathering phase if catatonia is present. To date, new inflammation CSF markers, such as IL-17A, IL-6, and chemokine profiles are only available for research purposes (29–31). Other mandatory paraclinical explorations include an electroencephalography (EEG) and a brain MRI. Additionally, PET scans are performed when the clinical presentation includes fever, neurological signs, and/or resistance to standard treatment in patients who have a negative MRI. A detailed list of these procedures and a proposed algorithm are part of a validated causality assessment score (CAUS) used to determine if an autoimmune condition may be related to the development of catatonia (32–34). Of note, the CAUS includes response to immunotherapy and high dose IV steroid challenge. Etiological treatments are classified into three groups: (1) tumor removal; (2) first line treatments, including IVIG, IV steroids and plasmapheresis; and (3) second line treatments, including cyclophosphamide, mycophenolate mofetil, azathioprine, and rituximab.

Despite differences in treatment approaches, all centers maintain an emphasis on early intervention even when autoimmune markers may be otherwise negative. While plasmapheresis is only available in three French centers, the use of plasmapheresis has now extended to all definite and probable AE when catatonia is present (34). Cyclophosphamide is mainly used in French centers. Mycophenolate mofetil or azathioprine is introduced after cyclophosphamide pulses (33). Rituximab is utilized in patients with definitive NMDA AE. Tumor removal in addition to other immunomodulatory treatments is completed when paraneoplastic processes are confirmed. New therapeutic options recently published, such as Tocilizumab and Il-2 are also considered in cases were symptoms are persistent despite first- and second-line therapies (24–26). At present, ECT is infrequently used and only considered in cases of resistant catatonia or malignant catatonia, regardless of etiology (35, 36).

## Discussion

There is a clear need for child and adolescent psychiatrists to become familiar with ways in which the clinical presentation of patients with autoimmune encephalitis may differ from a primary psychiatric disorder. Clinicians must then be able to articulate these differences to our multi-disciplinary colleagues in order to advocate for the diagnostic workup needed while still maintaining hypothesis flexibility. Randomized controlled studies dedicated to pediatric AE are lacking and without a clearly defined standard of care inequities will inevitably exist. Patients that are able to gain access to subspecialty and academic centers often receive care that is different from what may be available in more rural areas. Likewise, insurance barriers to care, geographic limitations, and access to treatment modalities within a particular health system may directly influence the care of the individual patient. Consensus is needed in order to develop an agreed upon standard of care that will facilitate access to diagnostic testing, timely transfer to a more specialized health system when indicated, and treatment options that may be otherwise considered novel.

Some programs offer risk stratification evaluations for patients who present with psychosis or catatonia regularly while other programs offer them on an infrequent basis. As an example, a 2020 position paper in the Lancet even proposed the concept of an autoimmune psychosis as a distinct clinical entity separate from autoimmune encephalitis (37). Additionally, some institutions are set up to mobilize complex multi-disciplinary care while others are relatively isolated in the care of patients without the potential benefit of consulting teams from neurology, immunology, infectious disease, or rheumatology. Moreover, some programs can offer immunomodulatory medications and ECT while others simply do not have these resources. In our provider network the most inconsistent resource for assessment is PET while the most inconsistent resource for treatment is ECT. Insurance barriers to care are notable in some health systems and this may influence the data available for comparisons within future international collaborative efforts. The treatment models outlined in this publication are merely a sampling of individual programs or hospital networks and the descriptions contained are not intended to dictate clinical care. It is our hope that this publication will facilitate dialogue surrounding practice norms internationally as opposed to focusing on a single health system.

Future research efforts may include a multi-institution standardized workup for all pediatric patients who present with possible AE. This would ostensibly provide valuable data regarding the predictive value of a given test or battery of tests. Acknowledging the current similarities and differences in practice norms as well as access to care may be a useful starting point for collaboration as inter-institutional and multidisciplinary research initiatives will be required in order to provide the highest level of care to our patients and their families.

## Data Availability Statement

The original contributions presented in the study are included in the article/supplementary material, further inquiries can be directed to the corresponding author/s.

## Disclosure

GM is an employee of the National Institute of Mental Health, Bethesda, MD. The views expressed in this article do not necessarily represent the views of the National Institutes of Health, the Department of Health and Human Services, or the United States Government.

## Author Contributions

GM served as the primary author and participated in the creation and editing of the manuscript. VF, ES, and DCF participated in the creation and editing of the manuscript. DCF participated in the manuscript editing and served as a content expert. All authors contributed to the article and approved the submitted version.

## Conflict of Interest

The authors declare that the research was conducted in the absence of any commercial or financial relationships that could be construed as a potential conflict of interest.
